# Hemorrhoids and cardiovascular disease: A bidirectional Mendelian randomization study

**DOI:** 10.1515/med-2025-1256

**Published:** 2025-08-19

**Authors:** Xin Ge, Weixin Tang, Jingmin Ni

**Affiliations:** Department of Traditional Chinese Medicine, Shanghai Chongming District Hospital of Traditional Chinese and Western Medicine, No. 2099 Honghai Highway, Chongming District, Shanghai, 202153, China; Department of Traditional Chinese Medicine, Shanghai Chongming District Hospital of Traditional Chinese and Western Medicine, Shanghai, 202153, China

**Keywords:** hemorrhoids, cardiovascular disease, Mendelian randomization, causal association, genetic analysis

## Abstract

**Background:**

Emerging evidence suggests that hemorrhoids are associated with cardiovascular disease (CVD). However, the causal associations between hemorrhoids and CVD remain elusive. This study aimed to investigate potential causal links between hemorrhoids and various heart conditions, including arrhythmia, heart failure, myocardial infarction, atrial fibrillation, and coronary artery disease.

**Methods:**

A two-sample bidirectional Mendelian randomization (MR) analysis was conducted using summary statistics of hemorrhoids and CVD from publicly available genome-wide association studies (GWAS). The MR analyses utilized inverse-variance weighted, weighted median, weighted mode, and MR-Egger methods. Sensitivity analyses included Cochran’s *Q* test, MR-Egger regression, MR pleiotropy residual sum and outlier (MR-PRESSO), and leave-one-out analysis. A radial MR analysis was performed after excluding outliers.

**Results:**

Genetically determined hemorrhoids did not exhibit a causal effect on arrhythmia (OR = 0.9998, *P* = 0.83), heart failure (OR = 0.94, *P* = 0.14), myocardial infarction (OR = 0.94, *P* = 0.27), atrial fibrillation (OR = 0.98, *P* = 0.55), or coronary artery disease (OR = 0.99, *P* = 0.84). The reverse analysis yielded similar results. Consistent results were observed with alternative MR methods, and the absence of significant heterogeneity was confirmed. The radial MR analyses support the conclusions in the forward and reverse analyses.

**Conclusions:**

This bidirectional MR analysis did not find statistical causal association between hemorrhoids and CVD, suggesting the possibility of shared risk factors such as obesity and diet. Further prevention strategies for CVD could focus on the management of common risk factors.

## 1 Introduction

Cardiovascular disease (CVD) stands as a leading global health challenge, encompassing a spectrum of conditions that affect the heart and blood vessels [1,2]. The epidemiology of CVD is marked by high prevalence and mortality rates, with coronary artery disease and heart failure being among the most prevalent forms [3,4]. The clinical features of CVD vary widely, from asymptomatic presentations to life-threatening events such as myocardial infarction and arrhythmia [5]. CVD is a multifaceted condition resulting from a complex interplay of various risk factors, such as age, sex, family history, hypertension, diabetes, smoking, and dyslipidemia [6,7]. The identification and understanding of additional risk factors for CVD are paramount for the development of effective strategies for both prevention and treatment.

Hemorrhoids, a common condition affecting the anal canal, are characterized by swollen vascular cushions that can cause discomfort and bleeding [8,9,10]. While typically considered a benign and manageable condition, hemorrhoids can significantly impact patients’ quality of life and prompt healthcare-seeking behaviors. This overt manifestation, which readily garners patients’ attention, contrasts sharply with the insidious nature of many CVDs, which often progress silently until advanced stages, posing a significant threat to life [11]. Several observational studies hinted at a possible association between hemorrhoids and CVD [12,13]. For example, Chang et al. showed that individuals with hemorrhoids have a 1.27-fold higher risk of developing coronary heart disease compared with those without hemorrhoids [12]. These findings, while intriguing, do not establish a causal relationship. Delving into the causal relationship between hemorrhoids, which manifest as a prominent symptom, and latent CVDs can significantly contribute to the effective management and prevention strategies for cardiovascular health.

Observational cross-sectional studies cannot be used to determine causality, and such studies are prone to residual confounding and a risk of reverse causality [14,15]. Longitudinal studies allow the determination of causality, but they remain prone to confounding and require extended follow-up durations [16], especially for slowly developing conditions like atherosclerotic diseases. Mendelian randomization (MR) is an innovative epidemiological approach that utilizes the random allocation of genetic variants from parents to offspring as a natural experimental setting to infer causality [17,18]. This method circumvents many of the biases inherent in observational studies, including confounding and reverse causality, offering a unique opportunity to explore the causal relationship between risk factors and disease outcomes [18,19]. In addition, MR studies use datasets from genome-wide association studies (GWAS) that often encompass tens of thousands of individuals. MR studies also allow the possibility of bidirectional analyses to determine the direction of causality.

This study aimed to investigate the causal association between hemorrhoids and a range of heart conditions, including arrhythmia, heart failure, myocardial infarction, atrial fibrillation, and coronary artery disease, using a two-sample bidirectional MR design.

## 2 Methods

### 2.1 Study design

**Figure 1:**
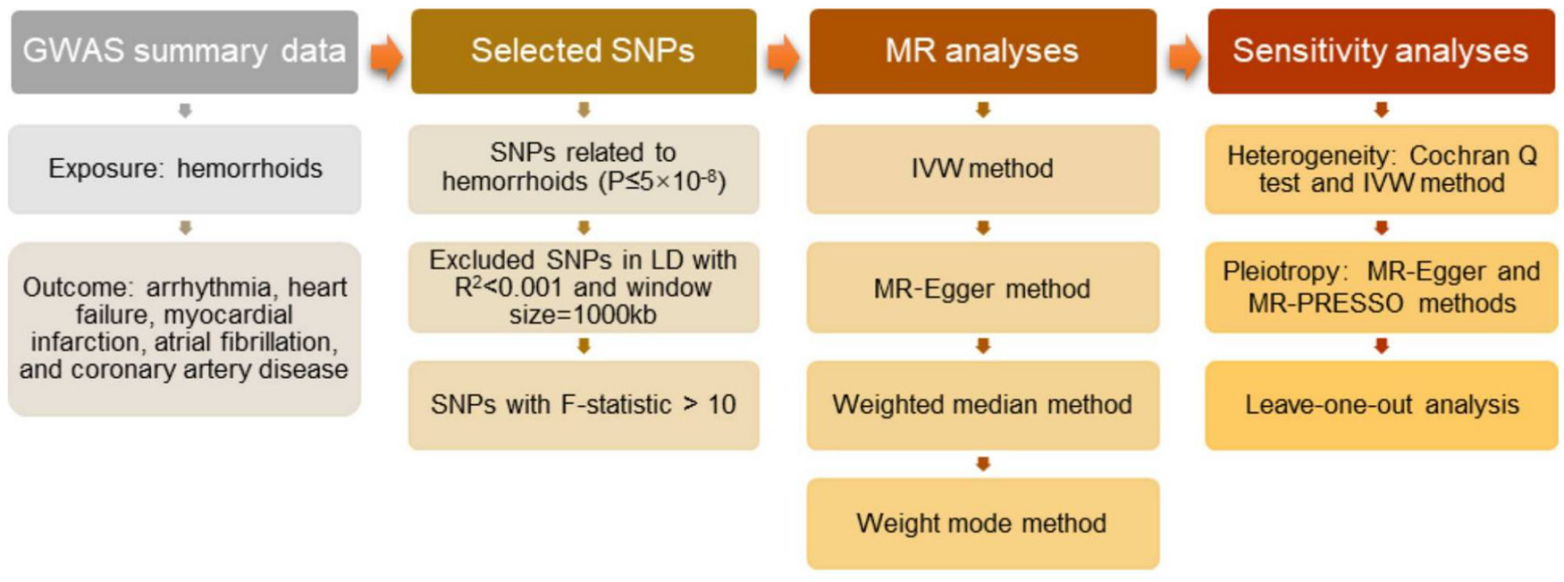
Workflow of the forward MR analysis revealing a causal relationship between hemorrhoids with CVDs. The same strategy was applied for the reverse analysis, but the outcomes and exposures were reversed. GWAS, genome-wide association studies; MR, Mendelian randomization; SNP, single-nucleotide polymorphism; IVW, inverse-variance weighted.

A schematic depiction of the MR design used in this study to delineate the potential causal relationship between hemorrhoids and CVDs is presented in Figure 1. We executed a two-sample bidirectional MR analysis, employing summary statistics extracted from GWAS, to probe into the causal association between hemorrhoids and CVDs. In this MR analysis, hemorrhoids were designated as the exposure of interest, with CVDs, including arrhythmias, heart failure, myocardial infarction, atrial fibrillation, and coronary artery disease, denoted as the outcome. Their roles were reversed in the reverse analysis. The foundational assumptions underlying the MR analysis were as follows: (a) the genetic variants are robustly associated with the exposure, ensuring a valid instrumental variable (IV) relationship; (b) the genetic variants are not confounded by factors that could potentially distort the relationship between exposure and outcome; and (c) the genetic variants exert their influence on the outcome solely through the exposure and not through any extraneous biological pathways, thereby maintaining the integrity of the causal inference [20].

### 2.2 Data sources

The GWAS summary data for hemorrhoids were obtained from the IEU GWAS database (https://gwas.mrcieu.ac.uk/). Furthermore, the GWAS summary data for various heart conditions, including arrhythmias, heart failure, myocardial infarction, atrial fibrillation, and coronary artery disease, were sourced from publications [21,22,23,24,25]. Specifically, the arrhythmia study encompassed a substantial 484,598 cases [21]. The heart failure study included 977,323 cases [22]. Myocardial infarction was the subject of a study that evaluated 14,825 cases alongside 44,000 controls [23]. Atrial fibrillation was explored in a study that featured 60,620 cases and 970,216 controls [24]. Finally, the coronary artery disease study involved 60,801 cases [25]. The detailed information for each dataset is presented in Table S1. This study is based on publicly available summary statistics, and no ethical approval is required.

### 2.3 IV selection

The approach to IV selection was predicated on a series of stringent criteria aimed at ensuring the reliability and validity of our genetic IVs. First, SNPs associated with the genome-wide significance of coronary artery disease were screened, that is, they met *P* < 5 × 10^−8^, and the remaining exposures met *P* < 5 × 10^−6^ because too few SNPs were identified using the more stringent threshold [26]. For the reverse analysis, all SNPs satisfied the *P* < 5 × 10^−8^ threshold. Then, SNPs with a minor allele frequency (MAF) > 0.01 were exclusively considered, a criterion that assured the SNPs’ representation in the population and their suitability as proxies for the genetic trait of interest [26]. A stringent LD exclusion criterion was applied to mitigate the potential confounding effects of linkage disequilibrium (LD). It involved maintaining *R* ^2^ < 0.001 within a defined window size of 10,000 kb, thereby preserving the independence and strength of the selected IVs [27]. In instances where an initially identified IV was absent from the outcome’s summary data, a high-LD proxy (*R* ^2^ > 0.8) was found that could effectively capture the genetic influence of the exposure. This step was crucial for ensuring the continuity of our genetic pathway analysis [26]. The strength of each IV was rigorously assessed using the *F*-statistic, a measure calculated as *F* = *R* ^2^ × (*N* − 2)/(1 − *R* ^2^), where *R* ^2^ represents the SNP’s proportionate contribution to the variability of the exposure within the IV. An *F*-value >10 was considered the threshold for confirming the IVs’ robustness [28,29].

### 2.4 MR analysis

The inverse-variance weighted (IVW) method was employed as the primary approach in the present study. Given the potential for heterogeneity between individual SNP causal estimates, the random-effect IVW model was utilized. This method is pivotal for interpreting MR findings, as it calculates the weighted average of the effect size, with the inverse variance of each SNP serving as the weight [30]. The odds ratio (OR) and its corresponding 95% confidence interval (CI) were determined to assess the causal relationship between the exposure and risk of the outcome under investigation. In addition, other complementary methods were used to enhance the robustness and reliability of the results. The MR-Egger method was used to address potential pleiotropic bias. This method acknowledges the presence of an intercept term and is adept at providing an accurate estimation of the causal effect even in scenarios where such bias is evident [31]. The weighted median method was also incorporated into this study. This approach operates under the assumption that at least half of the IVs are valid and is utilized to scrutinize the causal association between the exposure and the outcome [32]. All analyses were performed using R 4.0.5 along with the “Two-sample MR” package [33].

### 2.5 Sensitivity analysis

Cochran’s *Q* test was applied to assess the degree of heterogeneity among the IVs used in this study [34]. Taking into account the potential impact of the pleiotropic effects of genetic variation on the estimation of the association effect, the MR-Egger regression approach was adopted. Furthermore, the MR pleiotropy residual sum and outlier (MR-PRESSO) method was implemented to identify and correct for any potential outliers among the SNPs (defined as those with a *P*-value <0.05). After the exclusion of such outliers, the causal association was re-estimated, thus addressing and mitigating the influence of horizontal pleiotropy [35]. The leave-one-out analysis was conducted to ensure the robustness and consistency of our findings, providing insight into the influence of each individual IV on the overall MR estimate and confirming the stability of the observed associations [36].

Radial MR analysis was employed both to identify influential outliers [37] and to provide robust causal estimates after their exclusion. SNPs identified as outliers in both IVW and Egger’s radial MR analyses were re-examined after eliminating the outliers. If there was no heterogeneity, the results were retained.


**Ethics approval and consent to participate:** This article is a Mendelian randomization study. The data for this study were obtained from publicly available databases and published literature data and do not require ethical approval and written informed consent.
**Consent for publication:** Not applicable.

## 3 Results

### 3.1 IV selection

For the forward analysis, utilizing MR analysis with hemorrhoids as the exposure, 93 IVs related to arrhythmia, heart failure, myocardial infarction, atrial fibrillation, and coronary artery disease were selected. The average *F*-statistic for these IVs was 54.27, with the minimum *F*-statistic being 29.64 and the maximum reaching 203.18 (Tables S2 and S3). When arrhythmia, heart failure, or myocardial infarction served as the outcome, all SNPs were successfully matched with corresponding information in the summary data. However, for atrial fibrillation, one SNP could not be matched with the summary data, and no suitable proxy SNP was located. A similar issue arose with coronary artery disease as the outcome, where one SNP was unmatched, without a proxy SNP. For the reverse analysis, utilizing MR analysis with arrhythmia, heart failure, myocardial infarction, atrial fibrillation, and coronary artery disease as the exposures, 12, 111, 41, 12, and 78 SNPs were initially identified, respectively, with mean *F*-values of 85.08, 83.78, 61.28, 41.50, and 65.98, respectively (Table S4).

### 3.2 Causal effect of hemorrhoids on CVD

The MR analyses demonstrated that, following adjustment for multiple variables, there were no statistically significant associations between hemorrhoids and the risks associated with arrhythmia (OR = 0.9998, 95% CI: 0.9979–1.0017, *P* = 0.83), heart failure (OR = 0.94, 95% CI: 0.87–1.02, *P* = 0.14), myocardial infarction (OR = 0.94, 95% CI: 0.83–1.05, *P* = 0.27), atrial fibrillation (OR = 0.98, 95% CI: 0.90–1.06, *P* = 0.55), and coronary artery disease (OR = 0.99, 95% CI: 0.88–1.11, *P* = 0.84) when employing the IVW method, as depicted in Table 1. These findings were supported by the MR-Egger, weighted median, and weighted mode methods (all *P* > 0.05).

**Table 1:** MR analysis of causal association between hemorrhoids and CVD

Exposure	Outcome	No. SNPs	Methods	OR (95% CI)	*P*
Hemorrhoids	Arrhythmia	93	IVW	0.9998 (0.9979–1.0017)	0.83
			MR-Egger	0.9989 (0.9928–1.0051)	0.74
			Weighted median	0.9989 (0.9963–1.0016)	0.43
			Weighted mode	0.9991 (0.9944–1.0039)	0.72
	Heart failure	93	IVW	0.94 (0.87–1.02)	0.14
			MR-Egger	0.86 (0.67–1.12)	0.27
			Weighted median	0.93 (0.84–1.03)	0.16
			Weighted mode	0.89 (0.7–1.14)	0.36
	Myocardial infarction	93	IVW	0.94 (0.83–1.05)	0.27
			MR-Egger	0.70 (0.48–1.02)	0.06
			Weighted median	1.02 (0.93–1.11)	0.72
			Weighted mode	1.05 (0.89–1.25)	0.57
	Atrial fibrillation	92	IVW	0.98 (0.90–1.06)	0.55
			MR-Egger	0.95 (0.72–1.24)	0.69
			Weighted median	0.99 (0.91–1.08)	0.90
			Weighted mode	1.03 (0.84–1.27)	0.75
	Coronary artery disease	92	IVW	0.99 (0.88–1.11)	0.84
			MR-Egger	0.83 (0.56–1.23)	0.35
			Weighted median	0.96 (0.86–1.08)	0.50
			Weighted mode	0.88 (0.72–1.08)	0.22

A sensitivity analysis was conducted to verify the reliability of the IVW results. The MR-Egger regression analysis indicated that the study findings were not confounded by horizontal pleiotropy (all *P* > 0.05, Table S5). The Cochran’s *Q* test combined with the IVW method revealed that there was heterogeneity for the analyses of hemorrhoids and heart failure (IVW: *Q* = 133.1149, *P* = 6 × 10^−5^), myocardial infarction (IVW: *Q* = 409.6819, *P* = 0), atrial fibrillation (IVW: *Q* = 242.6223, *P* = 0), and coronary artery disease (IVW: *Q* = 239.7280, *P* = 0). In contrast, no such heterogeneity was observed between hemorrhoids and arrhythmia (IVW: *Q* = 103.3811, *P* = 0.12557) (Table S5).

In addition, the MR-PRESSO analysis identified some specific outliers. For heart failure as the outcome, three outliers were identified. When considering myocardial infarction, the analysis revealed nine outliers. Atrial fibrillation showed four outliers, and coronary artery disease had six outliers (Table S6). Significantly, upon the exclusion of these outliers, the association between hemorrhoids and these heart conditions continued to be non-significant (Table S7).

**Figure 2:**
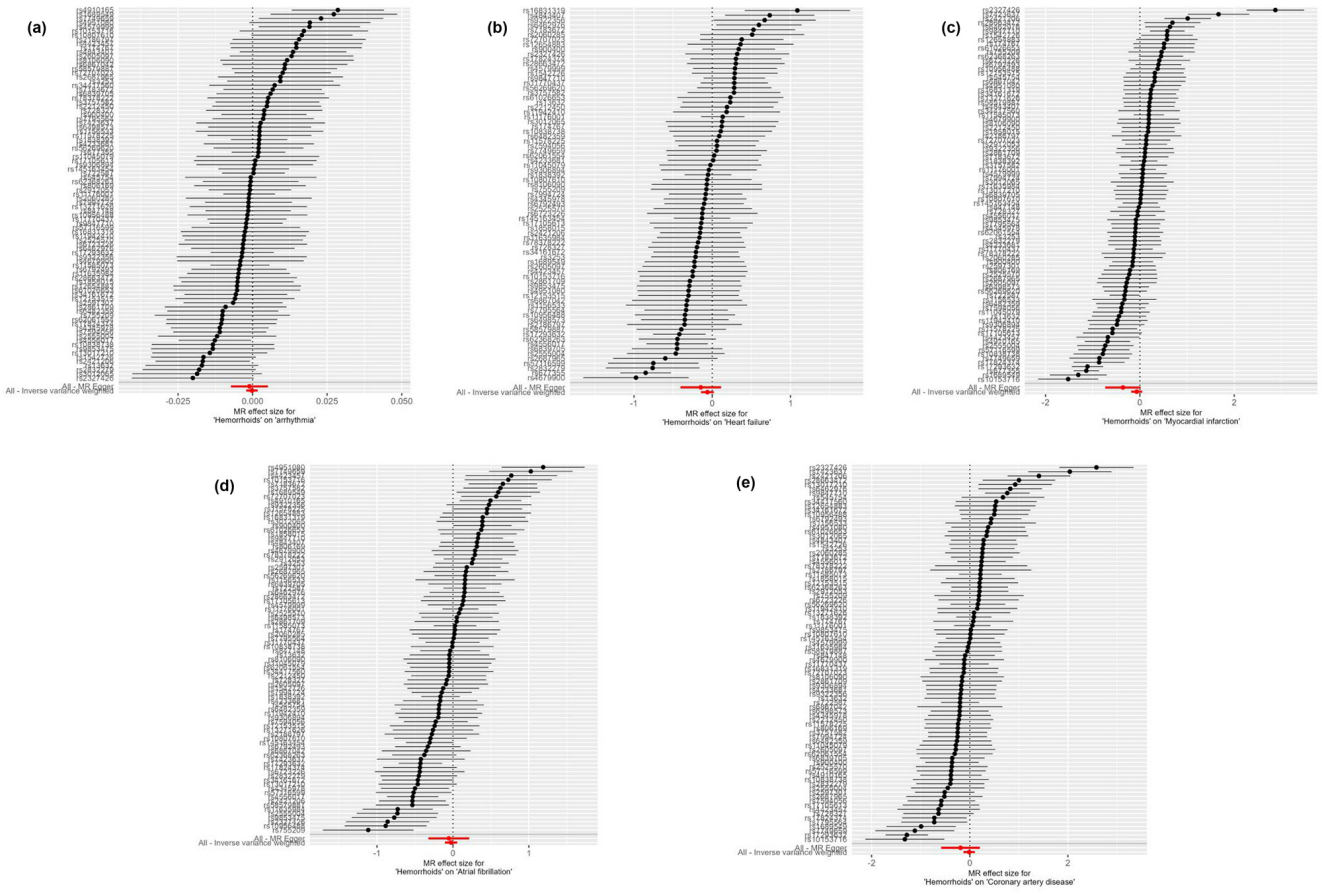
Forest plot of the MR effect size for determining the potential relationship between hemorrhoids with CVDs, including arrhythmia (a), heart failure (b), myocardial infarction (c), atrial fibrillation (d), and coronary artery disease (e). MR, Mendelian randomization.

**Figure 3:**
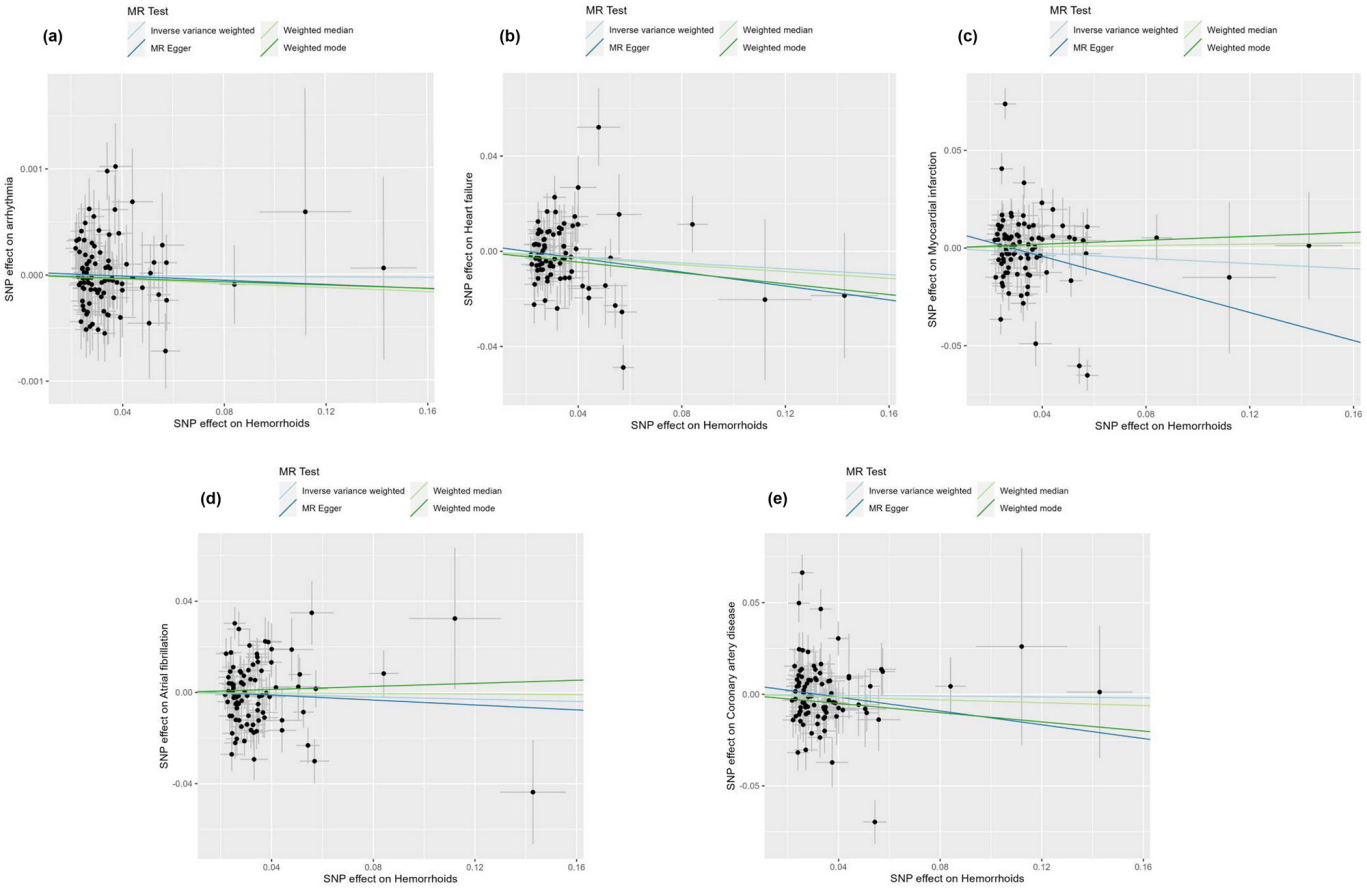
Scatter plot of the four MR models for determining the potential relationship between hemorrhoids with CVDs, including arrhythmia (a), heart failure (b), myocardial infarction (c), atrial fibrillation (d), and coronary artery disease (e).

**Figure 4:**
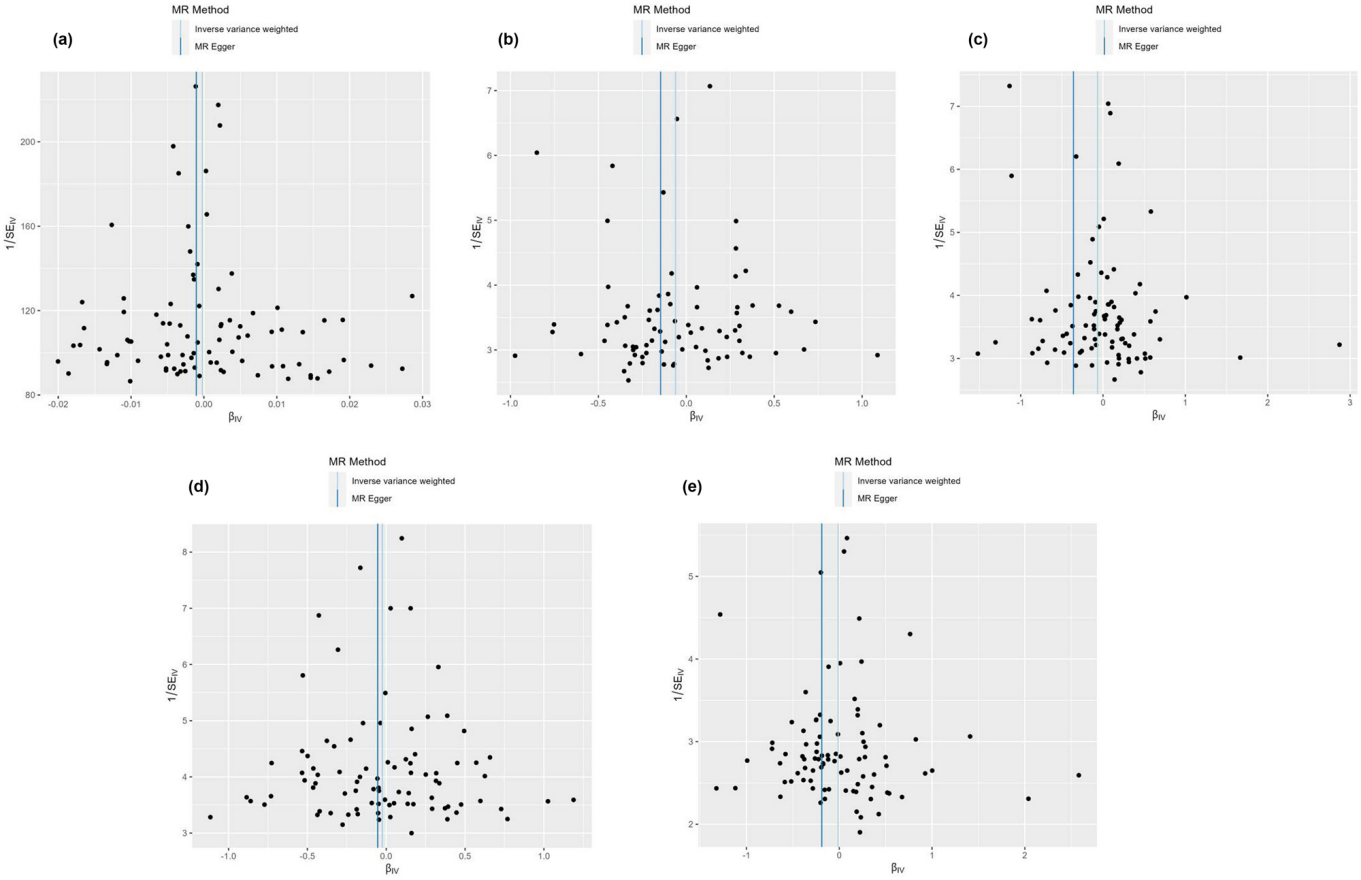
Funnel plot of the IVW model and MR-Egger model for determining the potential relationship between hemorrhoids with CVDs, including arrhythmia (a), heart failure (b), myocardial infarction (c), atrial fibrillation (d), and coronary artery disease (e).

**Figure 5:**
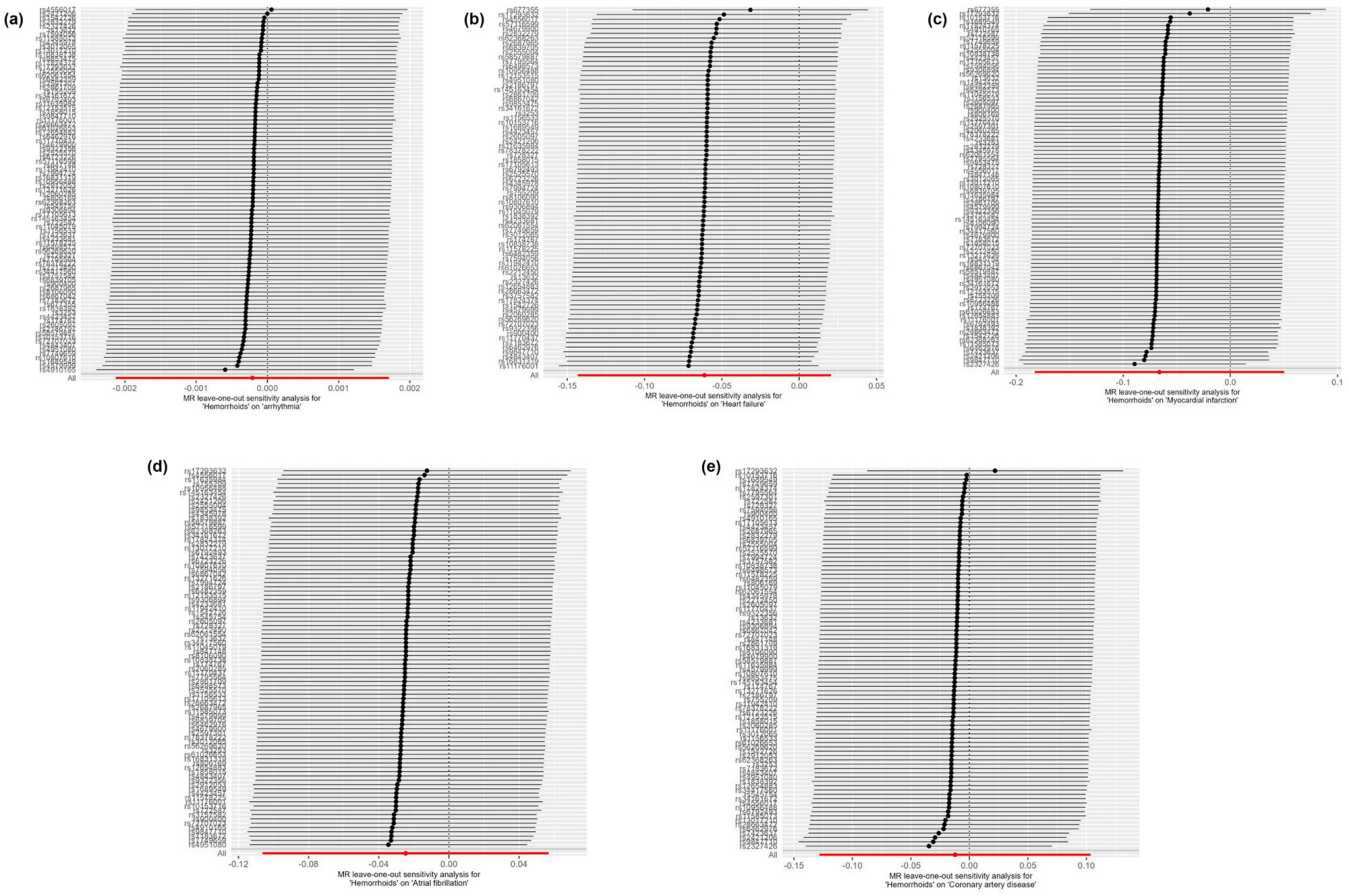
MR leave-one-out sensitivity analysis for determining the potential relationship between hemorrhoids with CVDs, including arrhythmia (a), heart failure (b), myocardial infarction (c), atrial fibrillation (d), and coronary artery disease (e).

The forest plot, as shown in Figure 2, features horizontal solid lines that correspond to the estimated results derived from individual SNPs. The visual representations of the causal associations from the MR analysis are presented in Figure 3. The funnel plot (Figure 4) demonstrates a symmetrical distribution around the effect estimate line. Additionally, the robustness of the findings was confirmed by sensitivity analyses using the leave-one-out method (Figure 5), which suggested that the above findings were robust, even when excluding the contribution of each SNP.

**Figure 6:**
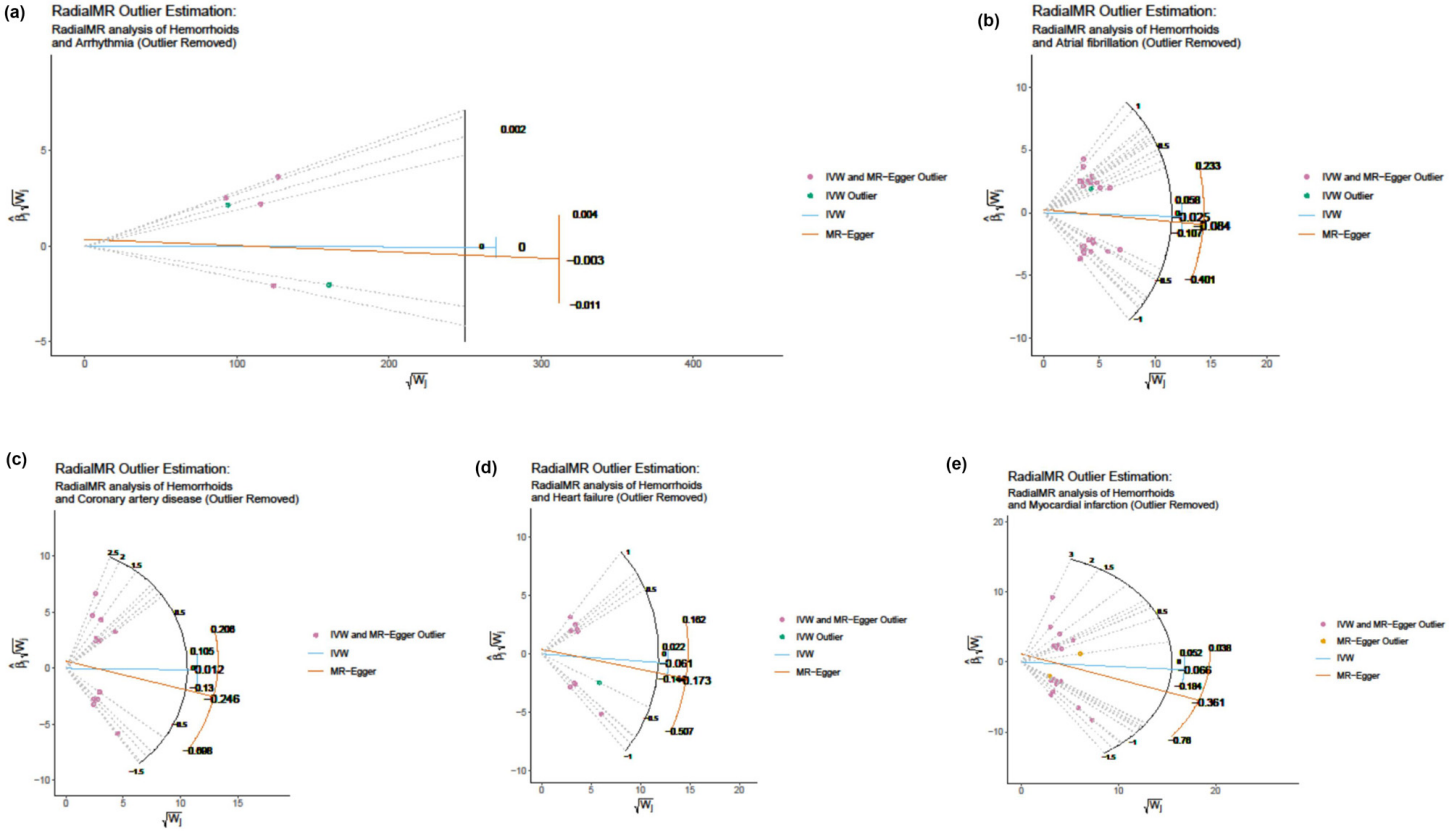
Egger radial MR analyses of the impact of hemorrhoids on arrhythmia (a), heart failure (B), myocardial infarction (c), atrial fibrillation (d), and coronary artery disease (e).

The genetic prediction results showed that after the SNPs identified as outliers in both the IVW and Egger radial MR analyses were excluded, there were no significant associations in all subsequent analyses, as shown in Table 2 and Figure 6. The heterogeneity test revealed that there was no heterogeneity in all analyses, and the results of the MR-Egger regression analysis indicated that all analyses were not affected by horizontal pleiotropy (Table S8). The MR-PRESSO analysis suggested that there are no outliers in the analysis, as shown in Table S9.

**Table 2:** Forward MR analysis (radial MR after eliminating outliers)

Exposure	Outcome	No. SNP	Method	OR (95% CI)	*P*
Hemorrhoids	Arrhythmia	85	IVW	0.9992 (0.9974–1.0010)	0.382
Hemorrhoids	Arrhythmia	85	MR Egger	0.9986 (0.9930–1.0043)	0.632
Hemorrhoids	Arrhythmia	85	Weighted median	0.9989 (0.9963–1.0015)	0.405
Hemorrhoids	Arrhythmia	85	Weighted mode	0.9990 (0.9944–1.0036)	0.671
Hemorrhoids	Atrial fibrillation	66	IVW	0.9797 (0.9236–1.0392)	0.496
Hemorrhoids	Atrial fibrillation	66	MR Egger	0.9790 (0.8121–1.1802)	0.825
Hemorrhoids	Atrial fibrillation	66	Weighted median	0.9977 (0.9181–1.0841)	0.956
Hemorrhoids	Atrial fibrillation	66	Weighted mode	1.0540 (0.8683–1.2796)	0.596
Hemorrhoids	Coronary artery disease	75	IVW	0.9632 (0.8928–1.0392)	0.333
Hemorrhoids	Coronary artery disease	75	MR Egger	1.0515 (0.8182–1.3514)	0.696
Hemorrhoids	Coronary artery disease	75	Weighted median	0.9606 (0.8587–1.0745)	0.482
Hemorrhoids	Coronary artery disease	75	Weighted mode	0.8342 (0.6536–1.0648)	0.150
Hemorrhoids	Heart failure	68	IVW	0.9510 (0.8902–1.0159)	0.136
Hemorrhoids	Heart failure	68	MR Egger	0.9114 (0.7414–1.1204)	0.382
Hemorrhoids	Heart failure	68	Weighted median	0.9338 (0.8422–1.0354)	0.194
Hemorrhoids	Heart failure	68	Weighted mode	0.8929 (0.6956–1.1462)	0.377
Hemorrhoids	Myocardial infarction	71	IVW	0.9882 (0.9293–1.0507)	0.704
Hemorrhoids	Myocardial infarction	71	MR Egger	1.0120 (0.8310–1.2325)	0.906
Hemorrhoids	Myocardial infarction	71	Weighted median	1.0262 (0.9394–1.1209)	0.567
Hemorrhoids	Myocardial infarction	71	Weighted mode	1.0700 (0.8950–1.2792)	0.460

OR: Odds ratio; CI: Confidence interval; and FDR: False discovery rate.

### 3.3 Causal effect of CVD on hemorrhoids

**Figure 7:**
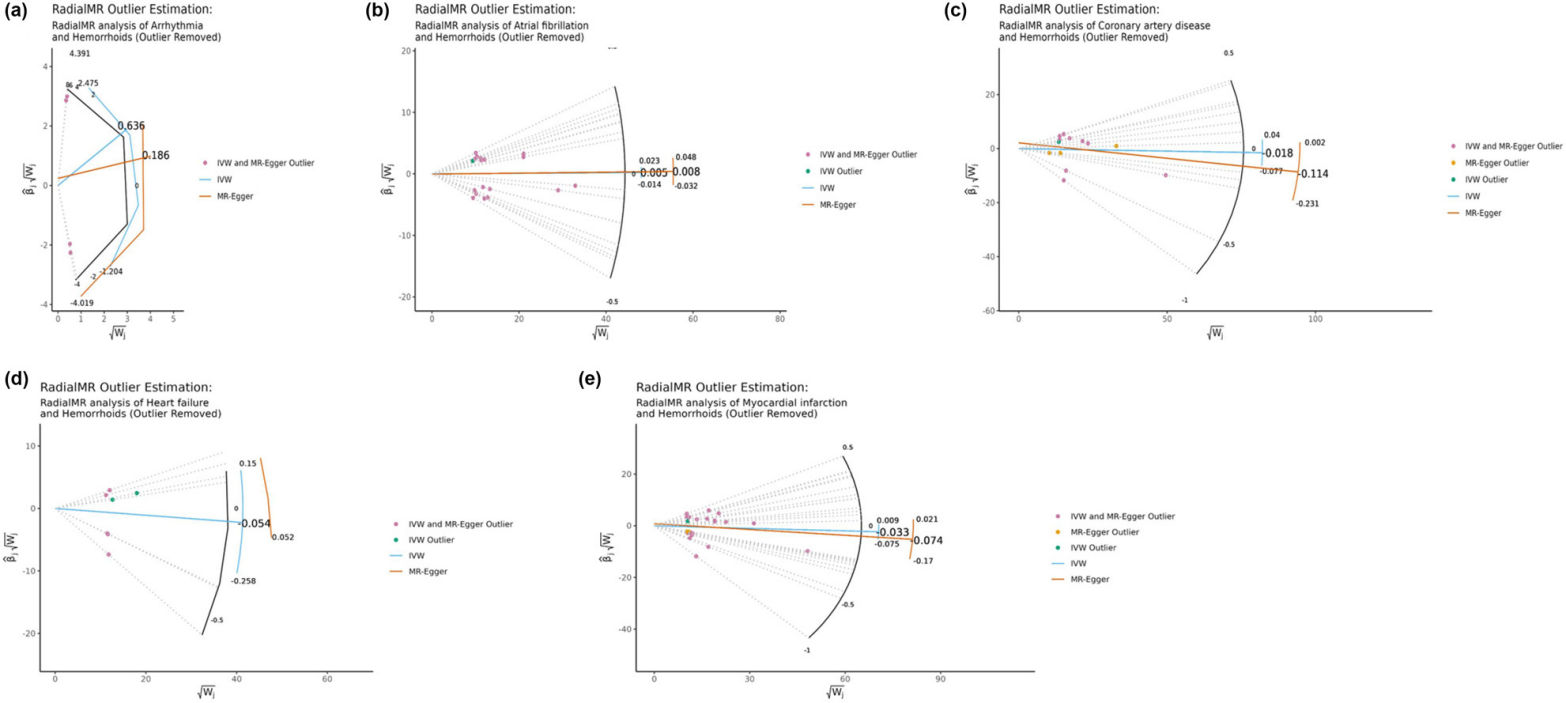
Egger radial MR analyses of the impact of arrhythmia (a), heart failure (b), myocardial infarction (c), atrial fibrillation (d), and coronary artery disease (e) on hemorrhoids.

The genetic prediction results showed that after the SNPs identified as outliers in both the IVW and Egger radial MR analyses were removed, there were no statistically significant associations in all analyses (Table 3 and Figure 7). Cochran’s test revealed no heterogeneity in all analyses. MR-Egger regression indicated the absence of horizontal pleiotropy as well (Table S10). MR-PRESSO suggested the absence of outliers in the analysis (Table S11).

**Table 3:** Reverse MR analysis (radial MR after eliminating outliers)

Exposure	Outcome	No. SNPs	Method	OR (95% CI)	*P*
Arrhythmia	Hemorrhoids	8	IVW	2.2542 (0.7602–6.6843)	0.143
Arrhythmia	Hemorrhoids	8	MR Egger	3.4228 (0.4503–26.0182)	0.279
Arrhythmia	Hemorrhoids	8	Weighted median	2.3358 (0.6431–8.4839)	0.197
Arrhythmia	Hemorrhoids	8	Weighted mode	2.5545 (0.6344–10.2865)	0.228
Atrial fibrillation	Hemorrhoids	91	IVW	1.0095 (0.9960–1.0233)	0.168
Atrial fibrillation	Hemorrhoids	91	MR Egger	1.0222 (0.9921–1.0533)	0.153
Atrial fibrillation	Hemorrhoids	91	Weighted median	1.0137 (0.9916–1.0363)	0.227
Atrial fibrillation	Hemorrhoids	91	Weighted mode	1.0175 (0.9915–1.0442)	0.192
Coronary artery disease	Hemorrhoids	25	IVW	1.0228 (0.9992–1.0469)	0.058
Coronary artery disease	Hemorrhoids	25	MR Egger	0.9987 (0.9377–1.0636)	0.967
Coronary artery disease	Hemorrhoids	25	Weighted median	1.0426 (1.0086–1.0777)	0.014
Coronary artery disease	Hemorrhoids	25	Weighted mode	1.0539 (0.9868–1.1255)	0.131
Heart failure	Hemorrhoids	3	IVW	0.9368 (0.8381–1.0472)	0.251
Heart failure	Hemorrhoids	3	MR Egger	1.0370 (0.8233–1.3062)	0.810
Heart failure	Hemorrhoids	3	Weighted median	0.9209 (0.8019–1.0575)	0.243
Heart failure	Hemorrhoids	3	Weighted mode	0.8824 (0.7387–1.0540)	0.302
Myocardial infarction	Hemorrhoids	51	IVW	0.9851 (0.9649–1.0057)	0.155
Myocardial infarction	Hemorrhoids	51	MR Egger	0.9814 (0.9281–1.0377)	0.512
Myocardial infarction	Hemorrhoids	51	Weighted median	0.9903 (0.9611–1.0203)	0.522
Myocardial infarction	Hemorrhoids	51	Weighted mode	1.0241 (0.9609–1.0915)	0.467

OR: Odds ratio; CI: Confidence interval; and FDR: False discovery rate.

## 4 Discussion

This study utilized an MR approach to explore the potential causal relationships between hemorrhoids and CVD. Our investigation, encompassing IVW, MR-Egger regression, weighted median, weighted mode methods, and radial MR analysis, did not reveal a significant association between genetically determined hemorrhoids and the risk of CVD. The reverse analysis yielded no significant associations either. Although MR-PRESSO identified outliers for several CVD outcomes (heart failure, myocardial infarction, atrial fibrillation, and coronary artery disease), subsequent analyses excluding these outliers, including radial MR, consistently showed no significant causal association between hemorrhoids and these conditions. Furthermore, the lack of significant heterogeneity across IVs, as evidenced by Cochran’s *Q* test, along with the leave-one-out analysis, substantiated the robustness of the results.

Despite the absence of a statistically significant causal association between hemorrhoids and CVD in the present MR analysis, the biological plausibility of a relationship between these conditions remains a topic of interest. The retrospective cohort study by Chang et al., which utilized reimbursement claims data from the Longitudinal Health Insurance Database 2000 in Taiwan, included 33,034 patients with hemorrhoids and 132,136 matched controls. After applying a Cox model to estimate the development of coronary heart disease, they reported that patients with hemorrhoids had a 1.27-fold higher risk of coronary heart disease compared with those without hemorrhoids, even after adjusting for potential confounding factors over a 12-year follow-up period [12]. This suggests several biological mechanisms: a high-fat diet could increase hemorrhoid risk due to intra-abdominal pressure from bowel movements, and obesity, a known risk factor for hemorrhoids, may contribute through stress on rectal muscles [12,38,39,40]. These factors are linked to atherosclerosis, a significant contributor to CVD [41,42,43].

However, Loosen et al. observed an increased rate of hemorrhoid diagnosis with a decreased incidence of coronary heart disease in the year prior to a colorectal cancer diagnosis [13], indicating a potential complexity in the relationship between hemorrhoids and CVD. The contrasting results may be due to biases inherent in the use of reimbursement claims data by Chang et al., such as misclassification or incomplete comorbidity capture [12]. In contrast, Loosen et al.’s focus on a specific patient population – those diagnosed with colorectal cancer – could represent unique risk factors and behaviors not seen in the general population [13]. The differences between these observational studies and the present MR analysis could be influenced by the cardiovascular system’s complexity, unaccounted gene–environment interactions, or other genetic or epigenetic factors not captured in our MR analysis. Besides, it is speculated that hemorrhoids and CVDs share risk factors such as obesity and diet, which lead to the correlation between them [44,45]. These factors may contribute to the inconsistencies observed in the relationship between hemorrhoids and CVD across various studies. Nonetheless, the present study contributes to the growing body of literature on the interplay between overt and covert health conditions. It emphasizes the importance of rigorous epidemiological and genetic methods in disentangling complex relationships between phenotypes and underscores the need for further research to identify dominant hemorrhoid manifestations as predictors for latent diseases such as CVD.

Previous studies suggested that hemorrhoids, particularly those with internal prolapse, may be a marker for increased risk of CVDs [12,46]. In addition, hemorrhoids and CVDs can be influenced by similar lifestyle factors [12,46]. On the other hand, the reverse analysis performed here showed no causal association between CVDs as exposure and hemorrhoids as outcome. Hence, it is plausible that CVDs and hemorrhoids are both the product of similar risk factors instead of sharing a causal relationship. Confounding is an important source of misinterpretation found in observational studies, and it is often difficult to eliminate despite the use of statistical methods like multivariable adjustment. On the other hand, MR analyses allow the observation of causality at the genetic prediction level without interference from confounders [47].

A strength of the present study is the MR method, which capitalizes on the random distribution of alleles during gamete formation to estimate causal effects, thereby reducing bias. The large sample size and the use of multiple causality assessment methods enhance the reliability of the findings. It is also important to consider the limitations of this MR study. The predominantly European ancestry of the study population may limit the generalizability of the results to other ethnic groups. Additionally, the cross-sectional nature of the GWAS data employed in the analysis restricts the capacity to draw temporal inferences, and there is an inherent potential for unmeasured confounding factors that could impact the relationship between hemorrhoids and CVD. Finally, this study used a radial MR analysis [37], adding strength to the study results.

Our initial MR analyses revealed heterogeneity in the associations between hemorrhoids and several CVDs. While precisely identifying the source of this heterogeneity is challenging, several factors can contribute to it in MR studies. These typically stem from violations of IV assumptions or underlying mechanistic complexities, including genetic variants with small effects on the risk factor amplify biases from pleiotropy [20,48]. Ancestry-related confounding can increase heterogeneity (e.g., genetic variants associated with socio-environmental factors) [48,49]. Parental genotypes influence offspring outcomes independently of the child’s genotype (e.g., via behavioral or epigenetic inheritance) [48]. Trait heterogeneity can also be responsible, especially when the risk factor represents a composite entity with subcomponents exerting distinct effects [49,50]. Methodological and analytical factors can lead to heterogeneity, including overlap bias (genetic associations for exposure and outcome derive from non-overlapping cohorts), NOME violation (ignoring uncertainty in genetic-exposure associations inflates heterogeneity), and outliers (single variants with extreme causal estimates distort pooled analyses) [51,52]. Last but not least, biological complexity plays a central role in disease associations, including non-linear effects (risk factor impacts vary by dosage or subpopulation, as in a U-shaped relationship) and context-dependent effects (variants influence outcomes only under specific environmental conditions). However, the present study employed several methods to address and mitigate the impact of heterogeneity. The IVW random-effects model itself can accommodate some heterogeneity [53]. Furthermore, complementary MR methods with different assumptions about pleiotropy, such as weighted median and MR-Egger regression, were utilized [31,54]. Crucially, the MR-PRESSO method was applied to detect and remove outlier SNPs, and subsequent radial MR analyses on the filtered datasets showed no significant causal effects and resolved heterogeneity, strengthening the robustness of our null findings.

In conclusion, this MR analysis, including a radial MR analysis, does not support a causal association between hemorrhoids and CVD in either direction. As the understanding of CVD and hemorrhoid etiology continues to evolve, further research is necessary to disentangle the multifactorial influences of multiple factors on the risk and progression of hemorrhoids and CVD. Determining the common risk factors and the mechanisms shared by the two conditions may ultimately inform the development of novel preventive and therapeutic strategies for CVD and hemorrhoids, two conditions that pose significant challenges to global public health.

## Supplementary Material

Supplementary Table
